# Investigation of the Bacterial Contamination and Antibiotic Susceptibility Profile of Bacteria Isolated from Bottled Drinking Water

**DOI:** 10.1128/spectrum.01516-21

**Published:** 2022-01-19

**Authors:** Anas Abdullah Hamad, Mohamed Sharaf, Manhal Ahmed Hamza, Samy Selim, Helal F. Hetta, Waleed El-Kazzaz

**Affiliations:** a Department of Medical Laboratory Techniques, Al Maarif University College, AL Anbar, Ramadi, Iraq; b Department of Biochemistry, Faculty of Agriculture, Al-Azhar University, Cairo, Egypt; c Department of Biochemistry and Molecular Biology, College of Marine Life Sciences, Ocean University of China, Qingdao, People’s Republic of China; d Faculty of Medical Laboratory Sciences, Department of Medical Microbiology, Omdurman Islamic University, Omdurman, Sudan; e Department of Clinical Laboratory Sciences, College of Applied Medical Sciences, Jouf University, Sakaka, Saudi Arabia; f Department of Medical Microbiology and Immunology, Faculty of Medicine, Assiut University, Assiut, Egypt; g Molecular Microbiology Lab., Botany Department, Faculty of Science, Suez Canal University, Ismailia, Egypt; University of Massachusetts Amherst

**Keywords:** bacterial contamination, domestic bottled waters, antibiotic susceptibility

## Abstract

The purpose of this study was to assess the bacteriological quality in some domestic bottled waters marketed in Al Anbar Province of Iraq. In total, 120 samples were collected from 20 different domestic bottled water companies. The current study findings demonstrated that the positive total bacterial count for aerobic bacteria was 20 CFU/ml (16.6%) out of 120 samples. From 120 tested samples, coliform bacteria had a much lower count of 13 CFU/ml (10.8%). The bacteriological analysis tests of this study showed that the brand bottled water of Alhilwa had the highest mean of total bacterial count at 485 CFU/ml, followed by Alwafi and Araco, which found at mean of 283 and 196 CFU/ml, respectively. The other brands of bottled waters included Sawa and Izmir, which had given lower mean of bacterial count at 87 and 58 CFU/ml, respectively, while all other tested brands of bottled waters had zero content of total bacterial count. According to the biomedical tests and Vitek2 system employed for this study, the isolated bacterial species as contaminants in bottled waters were Escherichia coli, Pseudomonas aeruginosa, and Klebsiella pneumoniae. The results of this study showed that Pseudomonas aeruginosa was sensitive to all tested antibiotics, but the Escherichia coli was resistance to amoxicillin, azithromycin, ceftazidime, and cefixime. The Klebsiella pneumonia demonstrated sensitivity to all tested antibiotics except the cefixime. Therefore, antibiotics belonging to the types of penicillin, carbapenem, and quinolones can be considered the best medicine for treating infections caused by the bacteria diagnosed in this study. In conclusion, the findings of this study showed that some domestic bottled waters sold in markets and shops in Al Anbar Province have bacteriological contents that are within permitted ranges for Iraqi and WHO standards.

**IMPORTANCE** Researchers analyzed how lifestyle factors affect the overall health of people with bacterial infections from the water. The article describes significance of the research because many people do not have access to clean, safe drinking water where this water is essential to life, and many die of waterborne bacterial infections. So, the purpose of the article is to draw attention to the major factors of the most dangerous bacteria transmitted through water marketed in Al Anbar Province of Iraq: Escherichia coli, Pseudomonas aeruginosa, and Klebsiella pneumoniae. Furthermore, our specific significant contribution has been to show the most important treatments for treating infections caused by the bacteria diagnosed in this study.

## INTRODUCTION

Access to safe drinking water is essential for survival. Safe water should not pose any danger to people at any stage of life, including children, the elderly, and vulnerable subpopulations such as immunocompromised people (1). For health maintenance, drinking water must be devoid of pathogenic bacteria, toxins, turbidity, odor, color, and taste (2). Despite different water disinfection, sanitation, and purification methods, waterborne diseases are still a major public health threat (3). According to the World Health Organization (WHO), contaminated drinking water causes 485,000 deaths annually. Contaminated drinking water might lead to waterborne diseases such as dysentery, diarrhea, cholera, typhoid, and polio. It is estimated that by 2025 half of the world's crowd will be living in water-stressed conditions (4). Infection following exposure to waterborne pathogens depends on many factors, including exposure dose, virulence, pathogen invasion, and host immune system (1).

By raising public awareness of waterborne diseases in recent years, bottled water is becoming an alternative to tap water all around the world (5). Water bottles are used by all people from different age groups with varying immunity states (6). Although bottled water is considered a safe alternative to tap water, it is not necessarily as safe as perceived. Several reports indicate that bacteria, viruses, and fungi in bottled water exceeded the standard limitations (5, 7). Thus, chemical or microbiological contamination can lead to severe gastrointestinal diseases (8). Bottled water contamination might result from contaminated water sources such as springs or during the bottling process from the environment, the equipment, and personnel (6).

A number of parameters should be assessed during monitoring water safety, as well as the total number of bacterial growths at 22°C and 37°C, total coliforms, fecal coliforms, and the presence of Escherichia coli and enterococci (8). However, several other pathogenic bacteria have been isolated from bottled water in different studies, including Pseudomonas aeruginosa (6), nontuberculous Mycobacteria (9), Salmonella spp., Vibrio cholerae (10), Klebsiella spp. (2), and Legionella pneumophila (11). Despite several reports on bottled water contamination in different countries, few studies have taken place in Iraq. Lack of enough information about the microbiological safety of bottled water in Iraq led to this study’s inception. This project aimed to evaluate the bacteriological quality of bottled waters in Al Anbar Province of Iraq using the culture, biomedical tests and Vitek2 system.

## RESULTS

### Isolation of bacterial species as contaminants from drinking bottled water.

**FIG 1:**
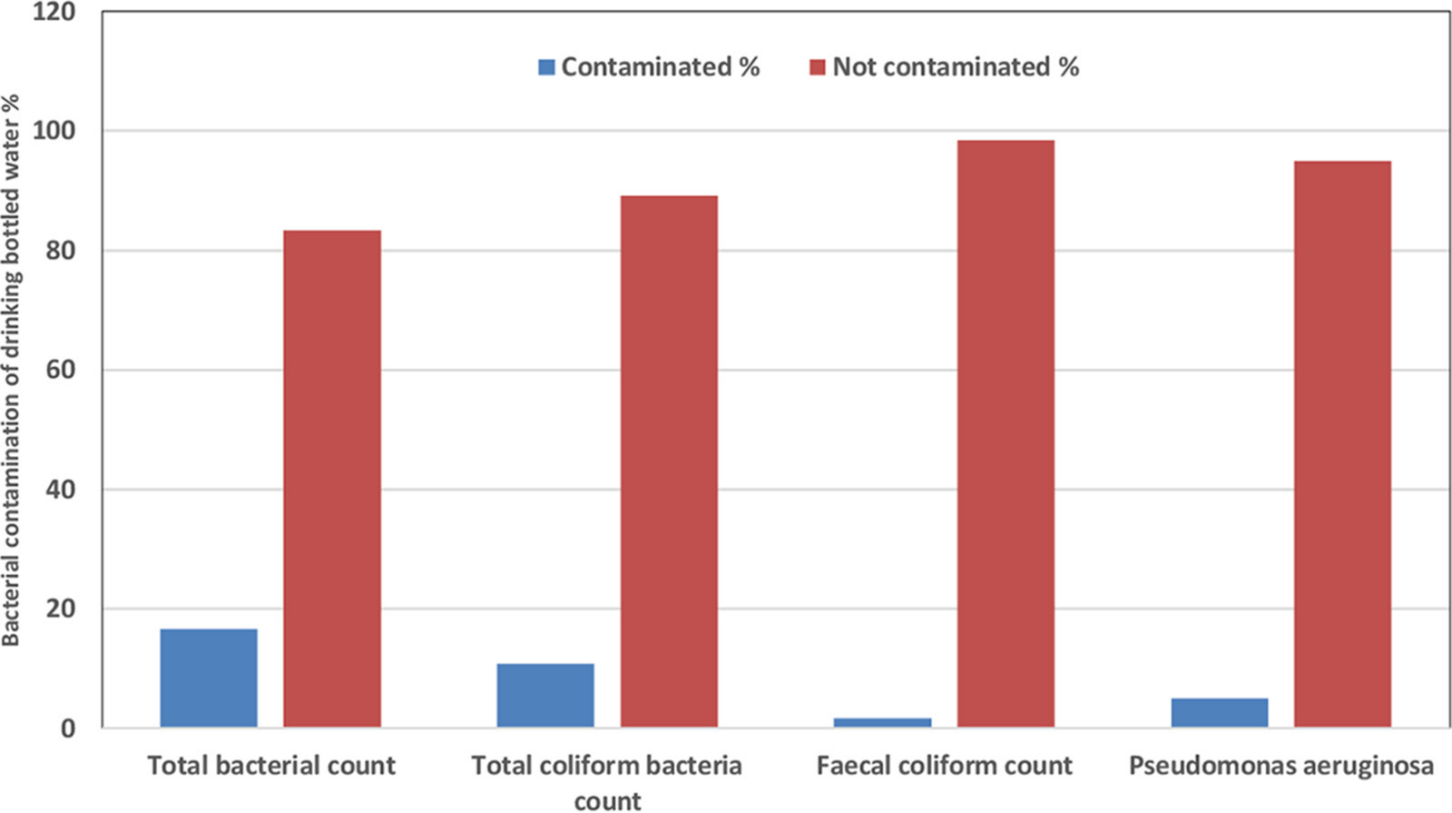
A variety of bacterial isolates were identified as contaminants in drinking bottled waters.

This study was carried out to assess the bacteriological quality of bottled waters which stored at different temperature ranged between 2–30°C and sold in different market areas of Al Anbar Province in Iraq. Table 1 shows the results of a total bacteriological count analysis of 120 different domestic bottled water samples representing the products of 20 different companies. The results of this investigation were ranged from zero to 485 CFU/ml as the mean for total bacterial count. The total bacterial count for the samples was found to be 20 (16.6%) as presented in Fig. 1, which is within the acceptable limits <20 CFU/ml. The highest mean of total bacterial count was recorded at 485 CFU/ml for bottled water brand Alhilwa, followed by Alwafi and Araco, which found at 283 and 196 CFU/ml, respectively. The other brands of bottled waters included Sawa and Izmir, which had giving lower mean of bacterial count at 87 and 58 CFU/ml, respectively. All five brands of contaminated bottled waters were stored at room temperature 30°C. Whereas all other tested brands of bottled waters were kept between 2–8°C and had zero content of total bacterial count as presented in Table 1.

**TABLE 1:** The total bacterial count of domestic bottled waters was determined through bacteriological analysis

Names of brands	The no. of samples tested	The no. & percentage tested samples for aerobic bacteria	Mean (CFU/mL)	SD
Alrawia	6	0 (0%)	0	0
Veneza	6	0 (0%)	0	0
Aquafina	6	0 (0%)	0	0
Peart	6	0 (0%)	0	0
Delta	6	0 (0%)	0	0
Izmir	6	2 (40%)	58	9.95
Alwafi	6	3 (60%)	238	103.07
Araco	6	4 (75%)	196	85.80
Sawa	6	5 (80%)	87	34.65
Alhilwa	6	6 (100%)	485	212.70
Royal	6	0 (0%)	0	0
Dijla	6	0 (0%)	0	0
Al Rayyan	6	0 (0%)	0	0
Affiat	6	0 (0%)	0	0
Nada	6	0 (0%)	0	0
Mizin	6	0 (0%)	0	0
Altuwq	6	0 (0%)	0	0
Al Hayat	6	0 (0%)	0	0
Barda	6	0 (0%)	0	0
Shaheen	6	0 (0%)	0	0
Total	120	20 (16.6%)		

The prevalence of total coliform bacteria was found to be at 13 (10.8%) in 100 ml of the tested samples. Five samples of Alhilwa out of six samples were contaminated with coliform bacteria with mean number being at 136 CFU/100 ml. Fig. 1 shows that fecal coliform bacteria were identified in two samples (1.6%) of Alhilwa brand. In addition, three samples of this brand were contaminated with Escherichia coli. Pseudomonas aeruginosa was detected in six samples (5%) from Sawa, Araco, and Izmir, with mean counts of 43, 38, and 14 CFU/100 ml, respectively. Only two of the six samples taken from Alwafi tested positive for Klebsiella pneumoniae, with a mean number of 32 CFU/100 ml, as presented in Table 2.

**TABLE 2:** The incidence of coliform bacteria isolated from domestic bottled water samples

Names of brands	The no. of samples tested	The no. & percentage positive for coliform bacteria	Mean (CFU/100mL)	SD
Alrawia	6	0 (0%)	0	0
Veneza	6	0 (0%)	0	0
Aquafina	6	0 (0%)	0	0
Peart	6	0 (0 %)	0	0
Delta	6	0 (0%)	0	0
Izmir	6	1 (20%)	14	12.32
Alwafi	6	2 (40%)	32	13.8
Araco	6	2 (40%)	38	10.86
Sawa	6	3 (60%)	43	16.63
Alhilwa	6	5 (80 %)	136	32.57
Royal	6	0 (0%)	0	0
Dijla	6	0 (0%)	0	0
Al Rayyan	6	0 (0%)	0	0
Affiat	6	0 (0%)	0	0
Nada	6	0 (0 %)	0	0
Mizin	6	0 (0%)	0	0
Altuwq	6	0 (0%)	0	0
Al Hayat	6	0 (0%)	0	0
Barda	6	0 (0%)	0	0
Shaheen	6	0 (0%)	0	0
Total	120	13 (10.8%)		

### Antimicrobial susceptibility testing.

Table 3 shows the findings of antimicrobial testing. The isolates, which related to Pseudomonas aeruginosa and Klebsiella pneumoniae, were found to be resistant to all antibiotics tested in this study. In contrast, the Escherichia coli isolates shown to be resistant to many antibiotics utilized included amoxicillin, azithromycin, ceftazidime, and cefixime.

**TABLE 3:** The antibiotics sensitivity findings against the bacterial species that isolated from drinking water bottles

Antibiotics		Bacterial species		
**Symbol**	**Name**	**E. coli**	**P. aeruginosa**	**K. pneumoniae**
PRL	Piperacillin	S^*a*^	S	S
AK	Amikacin	R	S	S
CFM	Cefixime	R	S	S
TOB	Tobramycin	S	S	S
CAZ	Ceftazidime	R	S	S
AZM	Azithromycin	R	S	S
AMC	Amoxicillin	S	S	S
CIP	Ciprofloxacin	S	S	S
CN	Cefalexin	S	S	S
IMP	Imipenem	S	S	S

a S = sensitive; R = resistant.

## DISCUSSION

The current study data revealed that the mean for isolated aerobic bacteria varied from 0 to 480 CFU/ml, with a total bacterial count of 20 (16.6%). The mean coliform bacteria count ranged from 0 to 136 CFU/100 ml, with a total bacterial count of 13 (10.8%). The reported results are not high when compared with WHO standard guidelines, which recommended a range of >20 CFU/ml. According to one study (12), the bacterial total count was determined at 36% of the drinking bottled waters samples, which is more than the WHO standard guidelines.

A research study has tested two different bottled waters including Salsal and Al Janaa en Al Mualaka in Basra city of Iraq and the bacteriological examination confirmed that no coliforms presence in two samples of bottled drinking water (13). The reason for not detected bacteria as contaminates in the bottled water, possibly related to the quality of the two companies for sterilization techniques utilized. In previous research (12), investigators tested seven different domestic brands of bottled water in Tehran, found that three (14.28%) out of the seven brands assessed were positive for Escherichia coli bacteria, indicating concern over the microbiological quality of bottled water and these results are in the line with my study.

Another study (14) has tested 20 different brands of bottled water which selected randomly at Jaipur city. The findings of this study have confirmed that out of 20, 50% of the samples were found insufficient in standard plate count. Several types of bacteria were detected including coliforms, E. coli and staphylococcal counts, which detected at 45%, 20%, and 5%, respectively. These results are relativity support the current study, as the total coliform bacteria in this investigation was found to be 10.8%.

Furthermore, two different studies have evaluated the quality of bottled waters. The first study has tested the domestic brands of bottled water in Hungary, and they found that out of the 246 noncarbonated mineral water samples examined, 187 (76.0%) had a 22°C below 100 CFU/ml, whereas at 37°C as many as 193 (78.4%) samples contained heterotrophic microorganisms at less than 20 CFU/ml (15). The second study has assessed the quality of the bottled waters in Dharan city, and the findings revealed that the bottled waters were positive for Pseudomonas spp. and Acinetobacter spp. at higher percentage of 87.5%, followed by *Citrobacter* spp. at 25% and the Chromobacterium violaceum was isolated at lower percentage of 12.5% (5). The results of the aforementioned studies are consistent with the current findings of this study. The current study findings indicated that the five brands of bottled water that corresponded to Izmir, Alwafi, Araco, Sawa, and Alhilwa were contaminated with various bacterial species, and this was potentially due to the storage temperature, as these brands were collected from room temperature 30°C. Furthermore, it is possible that the bacteria in the samples are viable but not culturable; further research is needed, and this may be a significant future topic to investigate.

The antibiotics outcomes of this study have showed that all drugs tested were effective against Pseudomonas aeruginosa and Klebsiella pneumoniae, while the amoxicillin, azithromycin, ceftazidime, and cefixime were among the antibiotics shown to be resistant in Escherichia coli isolates. The results of this study are comparable with those of other published studies (16, 17). In contrast, the outcome of this study contradicted with (18) study as the authors have reported that the Escherichia coli isolates showed sensitivity to cefixime, azithromycin, cefotaxime and ceftazidime. The difference can be explained by the fact that the isolation site as the cited study has isolated the Escherichia coli from wound infections whereas the current study from bottled water.

### Conclusion.

According to the findings of this investigation, some domestic bottled waters sold in markets and shops in Al Anbar Province have bacteriological contents that are within permitted ranges for Iraqi and WHO standards. Despite companies producing bottled drinking water and utilizing good sterilizing processes such as ozone and UV rays, they were not very effective in eliminating bacteria, and, as a result, several types of bacteria were observed. The health ministry is in responsible for supervising manufacturers to ensure that their work is closely monitored and that relevant health procedures are followed.

## MATERIALS AND METHODS

### Media preparation.

All culture media included nutrient agar, eosin methylene blue agar, and fecal coliforms agar base were prepared according to the instruction of manufacturing company. The media was sterilized by autoclaving at 121°C for 15 min.

### Samples collection.

During the period February 2021 to July 2021, a total of 120 samples of domestic bottled water (20 samples per month) regularly sold in Al-Anbar Province were randomly collected from several shops and markets in different locations of Al-Anbar Province. Overall, 120 samples were collected from 20 different domestic brands, with information about each brand provided in Table 4.

**TABLE 4:** Details of domestic bottled waters that have been tested

Names of brands	The no. of samples tested	Storage temp	The location of production	Disinfection methods
Izmir	5	30 C°	Al Anbar	Ozone & UV
Veneza	5	2–8 C°	Baghdad	Ozone
Aquafina	5	2–8 C°	Baghdad	Ozone & UV
Peart	5	2–8 C°	Baghdad	Ozone & UV
Delta	5	2–8 C°	Al Anbar	Ozone & UV
Alrawia	5	2–8 C°	Baghdad	Ozone & UV
Alwafi	5	30 C°	Baghdad	Ozone & UV
Araco	5	30 C°	Al Anbar	Ozone & UV
Sawa	5	30 C°	Babylon	Ozone & UV
Alhilwa	5	30 C°	Baghdad	Ozone & UV
Royal	5	2–8 C°	Baghdad	Ozone & UV
Dijla	5	2–8 C°	Baghdad	Ozone & UV
Al Rayyan	5	2–8 C°	Doha	Ozone & UV
Affiat	5	2–8 C°	Hilla	Ozone & UV
Nada	5	2–8 C°	Kirkuk	Ozone & UV
Mizin	5	2–8 C°	Baghdad	Ozone & UV
Altuwq	5	2–8 C°	Baghdad	Ozone & UV
Al Hayat	5	2–8 C°	Erbil	Ozone & UV
Barda	5	2–8 C°	Baghdad	Ozone & UV
Shaheen	5	2–8 C°	Pakistan	Ozone & UV

**Samples assessments.** All bottled water samples were tested for the following bacteriological criteria in accordance with WHO standards (19).

**Total bacterial count.** Both the pour plate and serial dilution techniques were used to perform the total bacterial count. After preparing serial dilutions of the bottled water in sterile normal saline, 1 ml of sample was transferred to a sterile, empty petri dish. Melting nutrient agar was added into the petri dish with the sample and thoroughly mixed. The plate was incubated at 37°C for 48 h after the mixture had solidified. The number of bacterial colonies that formed was assessed in CFU/ml.

**Total coliform bacteria count.** Using the membrane filter technique, total coliform bacteria count was measured. Each sample was filtered through a 0.45-μm pore size cellulose nitrate membrane filter (Sartorius, Germany) and then spread on eosin methylene blue agar (Himedia) and incubated at 37°C for 24 h.

**Faecal coliform count.** The number of fecal coliforms was measured using the membrane filter technique. One hundred milliliters from each sample was filtered through a cellulose nitrate membrane filter with a pore size of 0.45 μm and placed on fecal coliforms agar base (Himedia) for 24 h at 44.5°C.

### Identification of the bacterial isolates.

The bacterial isolates were identified using colony morphology, Gram staining, and biochemical characteristics. The biochemical tests utilized were catalase, oxidase, citrate utilization, urease, sulfide, indole motility, triple sugar iron test, methyl-red Voges Proskauer test, lysine decarboxylase test, slide coagulase test, tube coagulase test, and growth on bile esculin agar at 44.5°C. The colony morphology of some bacteria, such as fecal coliforms and total coliforms, in selective media like fecal coliforms agar base and eosin methylene blue agar, further aided in identification. The VITEK 2 system was used to perform additional identification for bacterial isolates. A vacuum device automatically filled the card, sealed it, and put it into the Vitek 2 reader-incubator module (incubation temperature 35.5°C), where it was exposed to kinetic fluorescence measurements every 15 min. The ID-GPC database interpreted the results, and the results were obtained automatically.

### Antimicrobial susceptibility testing.

Antimicrobial susceptibility testing was performed by disk diffusion method for all isolates using standard methodology (20). From a pure culture 3–5 selected colonies of bacteria were taken and transferred to a tube containing 5 ml of nutrient broth and mixed gently until a homogenous suspension was formed and incubated at 37°C until the turbidity of the suspension become adjusted to a McFarland 0.5. A sterile cotton swab was used, and the excess suspension was removed by gentile rotation of the swab against the internal surface of the tube. The swab was then used to distribute the bacteria evenly over the entire surface of Mullen Hinton agar. The inoculated plates were left at room temperature to dry for 5 min and a set of antibiotic disks, such as piperacillin (100 μg), amikacin (10 μg), cefixime (5 μg), tobramycin (10 μg) and ceftazidime (30 μg), azithromycin (15 μg), amoxicillin (30 μg), ciprofloxacin (10 μg), cefalexin (10 μg), and imipenem (10 μg), were dispensed on the surface of the inoculated Muller-Hinton plate.
